# An Experimental Method for Bio-Signal Denoising Using Unconventional Sensors

**DOI:** 10.3390/s23073527

**Published:** 2023-03-28

**Authors:** Rodrigo Aviles-Espinosa, Henry Dore, Elizabeth Rendon-Morales

**Affiliations:** Robotics and Mechatronics Systems Research Group, School of Engineering and Informatics, University of Sussex, Brighton BN1 9QJ, UK; r.aviles-espinosa@sussex.ac.uk (R.A.-E.);

**Keywords:** ECG, noise removal, Wiener, filtering, signal processing and electric field sensing

## Abstract

In bio-signal denoising, current methods reported in the literature consider purely simulated environments, requiring high computational powers and signal processing algorithms that may introduce signal distortion. To achieve an efficient noise reduction, such methods require previous knowledge of the noise signals or to have certain periodicity and stability, making the noise estimation difficult to predict. In this paper, we solve these challenges through the development of an experimental method applied to bio-signal denoising using a combined approach. This is based on the implementation of unconventional electric field sensors used for creating a noise replica required to obtain the ideal Wiener filter transfer function and achieve further noise reduction. This work aims to investigate the suitability of the proposed approach for real-time noise reduction affecting bio-signal recordings. The experimental evaluation presented here considers two scenarios: (a) human bio-signals trials including electrocardiogram, electromyogram and electrooculogram; and (b) bio-signal recordings from the MIT-MIH arrhythmia database. The performance of the proposed method is evaluated using qualitative criteria (i.e., power spectral density) and quantitative criteria (i.e., signal-to-noise ratio and mean square error) followed by a comparison between the proposed methodology and state of the art denoising methods. The results indicate that the combined approach proposed in this paper can be used for noise reduction in electrocardiogram, electromyogram and electrooculogram signals, achieving noise attenuation levels of 26.4 dB, 21.2 dB and 40.8 dB, respectively.

## 1. Introduction

Bio-signals are defined here as signals that originate from a human body [1]. These could be electrical, acoustic, mechanical or chemical. Examples of bio-electrical signal measurement devices include the Electrocardiogram (ECG), Electromyogram (EMG) and Electroocoulogram (EOG), which are widely used to support patient monitoring during surgical interventions or during intensive care unit (ICU) stays as well as for the development of wearable devices. However, the fidelity of the bio-electrical signals is often degraded by location-specific noise, which might alter the morphological features and the time interval aspects of the signal leading to false diagnosis and inadequate patient treatment.

The human electrocardiogram (ECG) provides information on the heart’s electrical activity using electrodes placed in contact with the skin. The ECG signal is represented by a non-linear quasi-periodic time series, and it is the key indicator to examine the electrical function and condition of the heart. However, the fidelity of the ECG signals is often degraded by noise, which might alter their morphological features (P, Q, R, S, T waves) and time dependent metrics such as the heart rate variability. Real time assessment techniques derived from the analysis of the ECG, EMG and EOG data rely on the identification of morphological and time interval characteristics. 

Applications such as cardiorespiratory monitoring and analysis of ECG and heart rate variability require accurate measurements and interpretation to aid and support diagnostics. Common sources of noise such as base-line wander (BW), muscle artefacts and power-line interferences (PLI) can severely affect ECG measurements [2], leading to inaccurate diagnosis and treatment [3].

ECG denoising methods have been classified into six main groups, according to [2,3]. The first group uses empirical mode decomposition (EMD), which is a local and adaptive method in frequency–time analysis. EMD is a data-driven mechanism which is suited to non-linear and non-stationary signals [4,5]. The second group includes deep learning based autoencoder models (DAEs), which aim at regenerating a clean ECG trace from a corrupted version of the same signal by optimizing the objective function. The wavelet-based methods fall into the third group. These use the wavelet transform (WT) to decompose the signal and determine the type of thresholding required to proceed with the signal reconstruction. The DAEs and WT are statistical methods and are used to extract a statistically-based model of the noise signal [6]. The fourth group utilizes the sparsity property of ECG for sparse optimization to denoise ECG signals. Here, the signal is split into segments; each one of these is broken into sparse parts and residues to denoise the ECG signals [7]. 

Even though several denoising approaches have been established in the literature, studies show that a single signal processing technique is not adequate to remove different sources of noise and artifacts present when collecting ECG signals [7]. Therefore, other advanced denoising approaches based on adaptive filtering have been proposed. The basic approach uses model-based Bayesian filters such as the extended Kalman filter (EKF), the Extended Kalman Smoother (EKS), and the unscented Kalman Filter (UKF). The fifth denoising group uses Bayesian filters to introduce changes in the conventional dynamic ECG model Kalman filter to denoise ECG signals. The last group is defined as a hybrid system combining various denoising methods previously reported in the literature [8]. It has been shown that combined approaches including filtering techniques such as conventional filtering [9] and adaptive filtering [10] can offer an improved reduction of noise in ECG signals. Various optimization techniques such as the total variation regularized least squares problem or a direct algorithm for 1-D total variation denoising (1DTVD) [10] have proved to be efficient alternatives for denoising ECG measurements. However, the results obtained using hybrid models showed that these filtering techniques introduce different kinds of waveform distortion [8]. Therefore, the development of a real-time, robust and reliable method to detect and remove the noise present in ECG recordings while preserving the signal fidelity still remains a challenge [3].

The state-of-the-art literature has also shown that Wiener filtering provides an effective method for noise reduction and ECG signal quality improvement. However, most of the denoising methods based on Wiener filtering rely on an estimation of the noise using a statistical approach [11,12]. Considering the known spectral properties (the power distribution of the signal in the frequency domain) within the signal of interest and the added noise, several computational iterations are required to approximate the transfer function of the Wiener filter in order to produce an output closely matching the signal of interest. Thus, ideal Wiener filtering requires having previous knowledge of either the noise level or the signal of interest, for effective noise removal. 

In this paper, a combined experimental approach using unconventional electric field sensors and Wiener filtering for bio-signal denoising is proposed. This new approach was inspired by a technique used in geophysical research [13]. 

This work aims to discover whether electric field unconventional sensors can be used for recording a real-time accurate noise replica to obtain the ideal Wiener filter transfer function resulting in optimum noise removal. Through the validation of this research question we aim to solve the challenges present in state-of-the-art denoising techniques, such as eliminating the requirement of noise signal estimation associated with the requirement of high computational powers, thus allowing real-time denoising while preserving the signal morphology. 

The proposed method is evaluated considering three cases of human bio-signals: (a) ECG, (b) EOG and (c) EMG measurements. The performance of the method presented in this paper is then compared with state-of-the-art methods using the MIT-BIH arrhythmia database. 

Experimental tests were carried out using a custom-made device for human bio-signal collection including a module that mimics the bio-electrical signal propagation based on tissue like phantoms. The details of the proposed methodology and the experimental tests are described in the following section.

## 2. Materials and Methods

The experimental method proposed in this work consists of a modification of the sinusoid subtraction method proposed by Legchenko [13] and subsequently improved by Larsen [14]. The Legchenko–Larsen method was originally used in geophysical magnetic resonance sounding measurements for the reduction of PLI. The authors propose a subtraction technique capable of suppressing stationary power-line noise. This technique has been shown to be effective in removing power line and short electromagnetic interference such as the characteristic bursts of cellular and Wi-Fi data transmission. The technique is based on subtracting an estimate of the harmonic component obtained from noise records and then combining with Wiener filtering. In this work, instead of estimating the noise, this is detected on site using unconventional electric field sensors in real-time. Subsequently, standard Wiener filtering is used to provide further noise reduction. 

### 2.1. Experimental Approach for Bio-Signal Denoising

The experimental denoising approach makes use of unconventional electric field sensors (EPS) [15]. EPS sensors, apart from providing high resolution ECG measurements, are susceptible to ambient noise, therefore making these ideal for detecting PLI and its harmonics including any other sources of external noise affecting the ECG recording.

EPS sensors fall within the category of capacitive sensing as they operate based on displacement currents. The structure of the EPS sensor has been previously described in [15,16]. Briefly, the EPS sensor is an electrometer-based amplifier with an insulating electrode that does not require galvanic contact with the body to acquire bio-potential signals. Instead, it operates with displacement currents where the traditional electrode-skin interface is replaced with a dielectric material.

**Figure 1:**
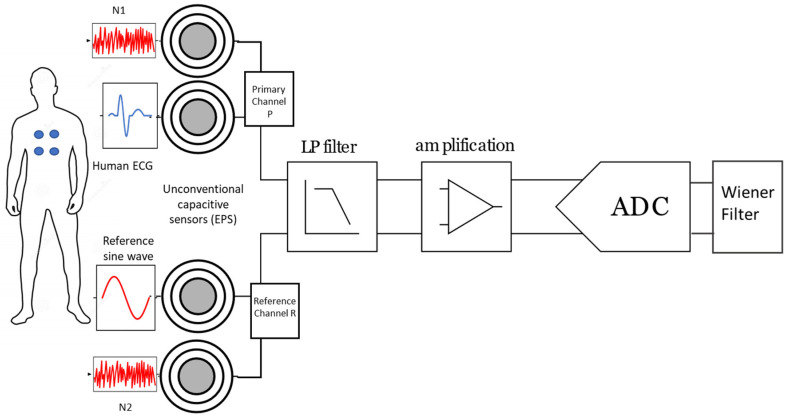
Experimental setup block diagram of the combined bio-signal denoising system configured for ECG detection.

The block diagram for the proposed bio-signal denoising system configured for ECG detection is shown in Figure 1. A two-channel ECG system was developed using unconventional electric field sensors [16]. The system consists of a primary channel (P), a reference channel (R) and two noise sources (N1, N2). The ECG signal with noise N1 was recorded using the primary channel. In the reference channel, a generated sine wave is used to capture the ambient noise N2. The signal pathways are assumed to be identical considering that the sensors are approximately positioned at the same distance from the noise source. Additional aspects such as sensor size, geometry and orientation are also considered to be identical. 

Both sets of sensors were connected to a signal conditioning stage for pre-filtering and amplification based on a data acquisition system (DAQ) built in a 16-bit analog-to-digital converter (ADS8319, Texas instruments, Dallas, TX, USA) for signal digitalization and a 16-bit digital-to-analogue converter (DAC8551, Texas instruments, Dallas, TX, USA) for signal output generation. Figure 1 also shows the Wiener filter functional block used to retrieve the ECG and noise signals from the measurements using the developed experimental sensing system. 

**Figure 2:**
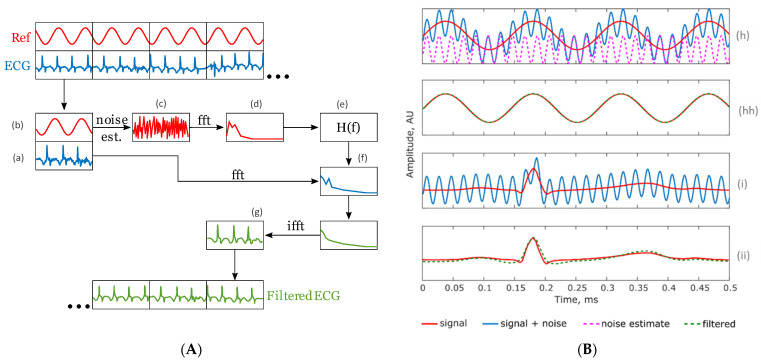
Details of the proposed bio-signal denoising approach. (**A**) Functional blocks and application of the Wiener filter. (**B**) Two example signals after applying the proposed denoising approach: (h) reference sine signal and noise replica (dotted line); (hh) reference sine signal after filtering; (i) idealized ECG signal highlighting QRS complex measured at the sensor’s output; (ii) ECG signal after filtering.

A detailed diagram of the proposed denoising approach applied to an ECG bio-signal including the main functional blocks and the Wiener filtering transfer function is shown in Figure 2. After recording both the noisy ECG (i.e., using the first pair of sensors) and sine waves (recorded using the second pair of sensors), the method works as follows. Firstly, the reference sine wave is subtracted from the output signal collected from the sensor to obtain the noise replica (see Figure 2A(c)). Secondly, the recorded ECG and noise replica are both fast Fourier transformed (FFT) at a sampling rate of 1 kHz giving a FFT frequency of 1 Hz (see Figure 2A(d)). Thirdly, based on the noise replica, the ideal Wiener filter transfer function is generated (see Figure 2A(e)) and then applied into the ECG signal (see Figure 2A(f)). Finally, the inverse FFT is applied to obtain the ECG signal with noise reduction (see Figure 2A(g)). 

Figure 2B shows an example trace after the denoising approach has been applied. This test was conducted using a function generator outputting two signals: a noise free 5 mV, 7 Hz reference sine wave and a replica with added 50 Hz noise. After applying the proposed method, the noise estimate was retrieved (see Figure 2B(h)) (pink dotted line). From this point, the ideal filter H(*f*) was calculated using Equation (2). The original and filtered sine waves can be compared in Figure 2B(hh), showing a minimal mean square error. Figure 2B(i) shows an idealized noise free ECG signal and the same signal with added 50 Hz noise (blue line). After applying the Wiener filter transfer function derived from the sine wave, the filtered ECG signal was obtained (see Figure 2B(ii)). It can be observed in Figure 2B(ii) that the 50 Hz noise is removed, with clear improvement in ECG signal quality.

### 2.2. Wiener Filtering

To obtain the ideal Wiener filter transfer function, the idealized ECG signal can be described as:

(1) $$x\left(t\right)=s\left(t\right)+n\left(t\right)$$

where *x*(*t*) is the ECG waveform recorded from the electric field sensor composed by the heart electrical signal *s*(*t*) and the noise component *n*(*t*). The optimal filter to reconstruct signal *s*(*t*) can then be calculated by:

(2) $$H\left(f\right)=\frac{P_{ss}(f)}{P_{ss}\left(f\right)+P_{nn}(f)}$$

where *H*(*f*) is the transfer function of the filter, *Pss*(*f*) and *Pnn*(*f*) are the known power spectral densities of the signal and noise, respectively.

## 3. Experimental Evaluation and Results

The proposed method was then validated employing the experimental setup shown in Figure 1. Testing was performed considering two scenarios: (a) attaching the sensors to the human body of a single participant to collect instant bio-signals including ECG, EMG and EOG; and (b) using ECG recordings from the MIT-MIH arrhythmia database.

For scenario (a), written consent was obtained from the participant before the study took place. The experiments were carried out in a private room offering a calm atmosphere to ensure the stability of the recordings. Measurements took place during a single session having the subject in the Fowler position. 

All the experiments were carried out following the experimental protocol approved by the University of Sussex Science and Technology Cross-Schools Research Ethics Committee (C-REC), adhering to the relevant guidelines and regulations. 

### 3.1. Results of Applying the Proposed Approach for Human Bio-Signal Denoising

**Figure 3:**
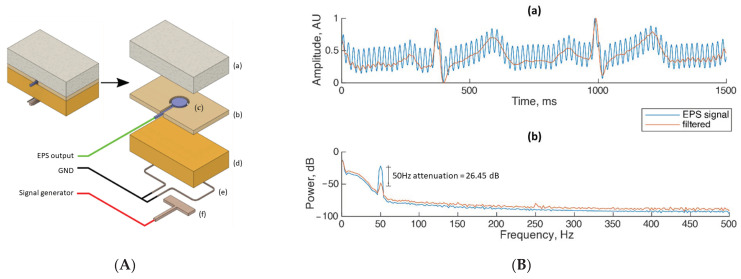
(**A**) The tissue phantom with an embedded electric field sensor. The stack consists of (a) foam block and weight, (b) insulating constraint layer, (c) electric field sensor, (d) agar phantom, (e) ground loop, (f) electric field source emitter. (**B**) Experimental results when applying the proposed denoising method on ECG bio-signals. (a) Human ECG signal collected using the sensors before (EPS signal) and after the denoising method is applied (filtered); (b) PSD measurements before and after the denoising method is applied.

For this proof-of-concept demonstration, two pairs of unconventional electric field sensors were placed on top of the participant’s chest, forehead, or wrist, respectively, for the collection of ECG, EOG, and EMG bio-electrical signals. The first pair of sensors were in contact with skin for bio-signal collection as shown in Figure 1. However, to avoid passing a sinusoidal electrical signal though the participant’s body, a phantom tissue was fabricated and added as an extra layer placed between the second pair of sensors and the human skin. The phantom tissue was used for recording the noise replica required as an input of the proposed method. Figure 3A shows the tissue phantom model that was fabricated within an embedded electrode to minimize variations in environment and ambient noise. It was placed near the subject’s skin to record the reference sine wave with a frequency of 7 Hz and an amplitude of 5 mV. The phantom tissue fabrication details have been reported in [17,18]. The predominant source of noise present in the laboratory environment was 50 Hz, sensed through the electric field sensors. 

#### 3.1.1. Case 1: ECG Testing

For the ECG experiment, the two pairs of unconventional electric field sensors were placed on the human chest as shown in Figure 1. The experimental results when applying the proposed denoising method to the collected human ECG signals are shown in Figure 3B. Figure 3B(a) shows a representative sample of a real-time ECG recording from the participant. The characteristic features of the ECG, such as the R peak, P and T waves, can be clearly distinguished. Although a residual amount of 50 Hz distortion is observed within the filtered signal, the noise is attenuated predominantly in the ST segment (the flat section of the ECG between ventricular depolarization and repolarization). Figure 3B(b) shows the recorded Power Spectral Density (PSD) plot where it can be observed that 50 Hz is the predominant source of noise without any important noise peaks present at the harmonic frequencies. After the method is applied, the 50 Hz noise is reduced by 26.45 dB. Across any of the additional frequencies within the power spectrum the signal is well correlated within both the filtered and raw traces, especially in the 1 to 40 Hz range where the ECG frequencies are located, indicating that no significant alteration to the ECG morphology is introduced by the proposed denoising method. 

#### 3.1.2. Case 2: EMG Testing

**Figure 4:**
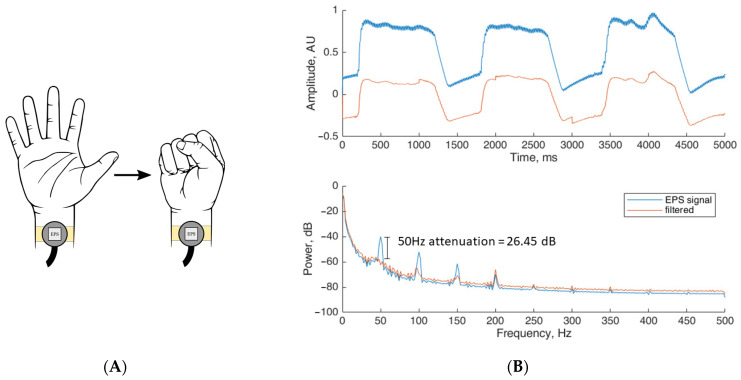
(**A**) Unconventional electric field sensors placed on the participant’s wrist for EMG bio-signal collection. (**B**) Experimental results when applying the proposed denoising method on EMG bio-signals. Figure 4B: (top) Human EMG bio-signal collected using the EPS sensors before (labelled EPS signal) and after the denoising method is applied (labelled as filtered); (bottom) PSD measurement before and after the denoising method is applied, showing an attenuation of 21.25 dB in the 50 Hz component.

For the EMG experiment, two pairs of unconventional electric field sensors were placed on the human wrist supported by a wristband as shown in Figure 4A. 

The phantom tissue was placed as described in Section 3.1 to record the noise replica required as an input of the proposed method. The participant was instructed to carry out a series of predefined hand movements repeatedly. During the test a fist was made and held for one second, followed by unclenching the hand for an additional second. The movements were repeated for a period of five seconds. The results obtained for the human EMG experiments are shown in Figure 4B. Figure 4B (top) shows a representative sample of the five second real-time EMG recording. A change in amplitude approximately every second is observed due to the change in the muscle contraction. In the same figure (red trace) is also observed a clear reduction of noise within the filtered signal. Figure 4B (bottom) shows the recorded PSD trace. Here an attenuation of 21.25 dB in the 50 Hz component is obtained after the method is applied. Across the power spectrum, the harmonic frequencies of 100 Hz and 150 Hz are attenuated by 20.9 dB and 11.4 dB, respectively. For the additional harmonic frequencies these remain unchanged, given their low magnitude. Finally, within the range of 1 to 40 Hz where the EMG signal is located, both the raw and filtered signals show good correlation, with minimal differences between them.

#### 3.1.3. Case 3: EOG Testing

**Figure 5:**
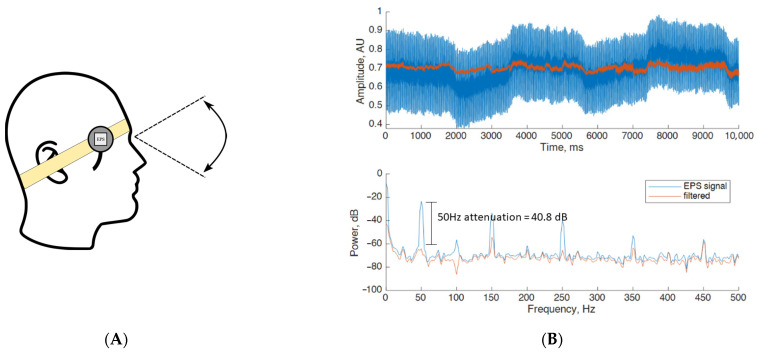
(**A**) Unconventional electric field sensors placed on the human forehead for EOG bio-signal collection. (**B**) Experimental results when applying the proposed denoising method on EOG bio-signals. Figure 4B: (**top**) Human EOG signal collected using the EPS sensors before (labelled EPS signal) and after (labelled filtered) the denoising method is applied; (**bottom**) PSD measured before and after the denoising method is applied.

For the EOG experiment, two pairs of unconventional electric field sensors were placed on the human forehead using a headband as shown in Figure 5A. The tissue phantom was placed as described in Section 3.1. The participant was instructed to carry out conjugate eye movements repeatedly for a total period of ten seconds. Experimental results when applying the proposed denoising method on the collected human EOG signals are shown in Figure 5B. Figure 5B (top) shows a representative sample of a ten-seconds real-time EOG recording. A step change in amplitude is observed due to the change in the eye position approximately every two 2 s. After applying the method in a similar way to the previous experiments, it can be observed that the filtered signal has a clear noise reduction. Figure 5B (bottom) shows the PSD trace having an attenuation of 40.8 dB around the 50 Hz frequency component. Across the harmonic frequency of 100 Hz, there is an important attenuation of 29.2dB, having the signal inverted with respect to the original trace. Despite this and given that the harmonic component magnitude is low when compared to the 50 Hz component, and the fact that it does not fall within the range of 1 to 40 Hz where the EOG signal is, there is no evident change in the signal morphology. Moreover, within the 150 Hz, 200 Hz, 250 Hz, 350 Hz and 450 Hz components, attenuations of 21.2 dB, 2.5 dB, 25.8 dB 10.8 dB and 2.5 dB are achieved, respectively Finally, in the range of 1 to 40 Hz, where the EOG power is located, minimum alteration between the filtered and raw data indicates that the proposed method is able to preserve the signal morphology.

### 3.2. Test Results of Applying the Proposed Approach Using MIT-BIH Arrythmia Database

The second test was designed to assess the proposed denoising approach effectiveness using the MIT-BIH arrhythmia database [19]. This repository contains standard test material commonly used for the evaluation of arrhythmia detectors. It contains 48 half-hour excerpts of two-channel ambulatory ECG recordings, obtained from 47 subjects. The database has been widely used in the literature for carrying out cardiac dynamics basic research [19,20] and is routinely used for ECG denoising and signal processing algorithm evaluation.

**Figure 6:**
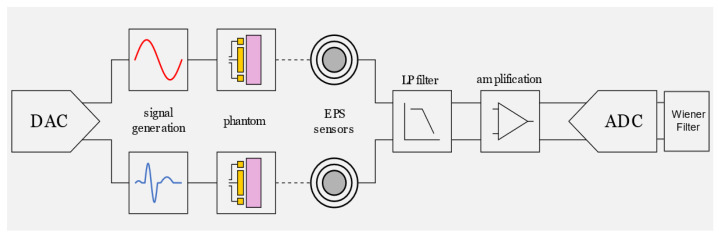
Block diagram of test system used to assess the proposed denoising approach using MIT-BIH database and tissue phantom.

Figure 6 shows the modified experimental set up used for the evaluation of the proposed denoising approach. The sine wave reference signals and simulated ECG signals (taken from the MIT-BIH database) were radiated using a pair of phantoms connected to the DAQ output. Two pairs of unconventional electric field sensors were placed in close proximity to the phantoms for collecting both the reference sinusoidal signal and the ECG signal including the ambient noise present in the lab.

As the MIT-BIH datasets were originally recorded at 360 samples/s, these where resampled to 1000 samples/s before the signals were radiated using the phantoms. The generated signals amplitude was 10 mV _P-P_ for both the sine reference wave and the MIT-BIH ECG bio-signals. Both signals were collected through a pair of sensors and processed using an analog front-end consisting of a 2nd order antialiasing low pass filter (cutoff frequency fc = 500 Hz) and an amplification stage. Digitization and signal generation was performed as described in section two. The collected data was then processed using MatLab. Two performance metrics were considered to evaluate the signal quality after the proposed denoising method was applied: (a) the mean square error (MSE); and (b) the 50 Hz attenuation value. The MSE was calculated using Equation (3), considering the averaged squared difference between the generated bio-signal and the resulting filtered signal.

(3) $$MSE=\frac{1}{n}\sum_{i=1}^{n}{\left(Y_{i}-{\hat{Y}}_{i}\right)}^{2}$$

where $Y_{i}$ is the filtered signal, ${\hat{Y}}_{i}$ is the generated MIT-BIH ECG bio-signal radiated though the antenna and phantom arrangement, considering n = 1000 for the 1 s window sampled at 1 kHz. 

The 50 Hz attenuation value is derived from the PSD measurements by subtracting the power at 50 Hz of both ECG bio-signals before and after applying the proposed denoising method, both available from the FFT of each time window. Both the MSE and 50 Hz attenuation values are averaged across all windows for each individual test to get a time window averaged MSE. 

For this experiment, 5-min samples considering the first 8 records (labelled m100 to m107) contained in the MIT-BIH ECG database were used. The mean values for both the MSE and 50 Hz attenuation were calculated for each 1 s window. The reference sine wave amplitude and phase were matched to the generated sine wave. The signals were then subtracted to obtain the noise replica. Then, the ECG and noise estimates were fast Fourier transformed employing a sampling rate of 1 kHz, giving a FFT frequency resolution of 1 Hz. 

**Figure 7:**
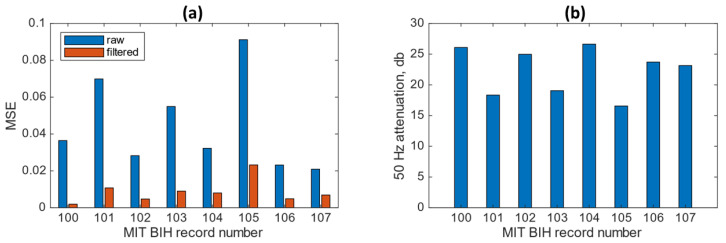
(**a**) MSE results applying the proposed denoising approach. (**b**) 50 Hz attenuation obtained from the MIT-BIH dataset records labelled m100 to m107.

Figure 7a shows the MSE results obtained from the MIT-BIH dataset records labelled m100 to m107 before and after applying the proposed denoising approach. The application of the combined approach shows a clear signal quality improvement in all the records, with a maximum reduction of 95% MSE for record m100 and a minimum of 67% for record m107. The averaged MSE across the filtered records was 0.0087. Figure 7b shows the 50 Hz attenuation values after applying the combined approach. The level of 50 Hz attenuation shows that all records have at least 16 dB of attenuation with an average across all records of 22.3 dB. Records m100, m102 and m104 show the highest attenuation values, corresponding to 27.2 dB, 25.8 dB and 27.3 dB, respectively.

To evaluate the performance of the combined denoising method, we compared the results obtained in this study with other related works focused on noise reduction using alternative state of the art denoising techniques such as Finite Impulse Response (FIR) [21], Normalized Least Mean Square (NMLS) [22], Empirical Mode Decomposition (EMD) [23] and Discrete Wavelet Transform (DWT) [24]. 

In [21], four filtering techniques, namely, DWT, NLMS, FIR and Infinite Impulse Response (IIR), were evaluated using denoising simulation tools applied to five MIT-BIH ECG datasets for PLI reduction. In [22], a modified adaptive algorithm introducing symbolic functions and block-processing concepts was implemented for the elimination of PLI in ECG signals. In [23], an ECG denoising method based on EMD was proposed, being able to remove high frequency noise and having a reduced signal distortion. In [24], a three step denoising algorithm was applied, based on the DWT decomposition, an adaptive dual threshold filter (ADTF) step and the highest peaks correction step for dealing with both the EMG noise and power line interference perturbing the ECG signal.

Table 1 shows a comparison of the above-mentioned noise reduction techniques using the MIT-BIH ECG database. It is worth mentioning that record 100 is widely used in the related works outlined previously [21,22,23,24] given that this first record in the database is used as a baseline for the comparison between different denoising approaches. The results obtained after applying the proposed methodology show that the resulting MSE value is comparable with the results obtained in the related works using FIR and NLMS [21,22]. When applying the experimental denoising approach proposed here, the SNR value shows improvement as compared to the EMD [23] and DWT [24] approaches.

## 4. Discussion 

The results obtained from the experimental work presented in this paper demonstrate that the proposed combined approach using unconventional electric field sensors is suitable for denoising PLI interference present both in the human body and in pre-recorded bio-signals including ECG, EMG and EOG. Through the subtraction of the recorded noise signals measured using a reference sine wave and electric field sensors, it is possible to obtain a noise replica and generate the ideal Wiener filter settings. This provides adaptative noise cancelling capabilities where both the original and filtered signals overlap within all the ECG characteristic segments presenting a PLI attenuation from −25 to −52 dB. 

Moreover, the harmonic frequencies present within the recorded signal are attenuated proportionally as observed in the EMG and EOG tests. This is shown in the PSD traces depicting the collected signal containing the noise present in the lab and the filtered version of it. It is evident from the traces that, in most of the cases, the filtering technique is able to attenuate such sources of noise without compromising the original signal morphology and to leave all its characteristic features intact, thus allowing these to be used in clinical diagnosis as well as HR calculation when dealing with ECG traces. 

The MSE results obtained from the MIT-BIH database, considering a total of 40 min of test data contained in multiple records, show an average reduction in the MSE of 80%. Here, 50 Hz PLI was attenuated on average by 22.3 dB in the tested datasets. Examination of the ECG waveforms and power spectral density components showed no significant alteration of the ECG morphology. 

Importantly, related works outlined in Table 1 are limited to evaluations conducted using simulation software to generate ECG signals adding simulated 50 Hz PLI. To the best of our knowledge, no comparable studies implementing an experimental approach have been reported in the literature. In contrast, this work presents an experimental methodology considering real-time human ECG containing ambient PLI noise detected though electric field sensors. The MSE is comparable with the results obtained in both the FIR filtering and NLMS methods, where the improvement in SNR is higher than that of either the EMD or DWT methods. This SNR improvement of 1.88 dB and 3.38 dB when comparing the here-presented method with the EMD [23] and DTW [24] denoising techniques may seem moderate; however, the combined experimental denoising method presented in this paper inherently introduces additional errors as it considers real noise collected when radiating the signal though the developed phantom in contrast with the ideal signals and simulated noise employed in the all the approaches outlined in Table 1. It can be strongly inferred that this method would have similar results in an environment where the mains frequency is 60 Hz and the associated harmonic frequencies. 

The obtained results serve to validate the research question formulated in this paper demonstrating that the method presented here is able to solve the challenges present in state-of-the-art denoising techniques, such as the requirement of noise signal estimation, and the requirement of high computational powers, allowing real-time denoising of bio-signals while preserving their morphology. 

## 5. Conclusions

In this paper, we have proposed a combined experimental approach using unconventional electric field sensors and Wiener filtering for bio-signal denoising. This new approach has been inspired by a technique used in geophysics to remove PLI and its harmonics in magnetic resonance sounding. This work provides improvements compared to the geophysical approach as the noise signal does not need to be estimated but instead, electric field unconventional sensors are used for recording an accurate replica of the sensed noise in real-time. By combining this with standard Wiener filtering, the method presented here can eliminate not only the 50 Hz powerline interference but also its harmonics without altering the morphology of the original bio-signal. Future work will be focused on evaluating the proposed approach using a multichannel ECG system based on unconventional electric field sensing in both a laboratory and a hospital setting.

## Figures and Tables

**Table 1:** Comparison of different filtering methods for MIT-BIH database record 100.

Method	References	Noise (Source)	SNR Improvement, dB	MSE
This work	-	50 Hz + WGN (ambient)	10.7	0.0019
Infinite impulse response (FIR)	[21]	WGN (simulated)	N/A	0.0018
Normalized least mean square (NLMS)	[22]	50 Hz (simulated)	N/A	0.002
Empirical mode decomposition (EMD)	[23]	WGN (simulated)	8.82	N/A
Discrete wavelet transform (DWT)	[24]	WGN (simulated)	7.32	N/A

## Data Availability

The data presented in this study are available on request from the corresponding author. The data are not publicly available due to biometric identity information. Restrictions apply to the availability of third-party data obtained from the MIT-BIH arrhythmia database and are available from the authors at URL: https://physio-net.org/physiobank/database/html/mitdbdir/intro.htm with the permission of [George B. Moody (george@mit.edu)].
